# A Model-Based Design Floating-Point Accumulator. Case of Study: FPGA Implementation of a Support Vector Machine Kernel Function †

**DOI:** 10.3390/s20051362

**Published:** 2020-03-02

**Authors:** Marco Bassoli, Valentina Bianchi, Ilaria De Munari

**Affiliations:** Department of Engineering and Architecture, University of Parma, Parco Area delle Scienze, 181/A, 43124 Parma, Italy; marco.bassoli@unipr.it (M.B.); ilaria.demunari@unipr.it (I.D.M.)

**Keywords:** model-based design, FPGA, HDL code generation, wearable sensors, embedded devices

## Abstract

Recent research in wearable sensors have led to the development of an advanced platform capable of embedding complex algorithms such as machine learning algorithms, which are known to usually be resource-demanding. To address the need for high computational power, one solution is to design custom hardware platforms dedicated to the specific application by exploiting, for example, Field Programmable Gate Array (FPGA). Recently, model-based techniques and automatic code generation have been introduced in FPGA design. In this paper, a new model-based floating-point accumulation circuit is presented. The architecture is based on the state-of-the-art delayed buffering algorithm. This circuit was conceived to be exploited in order to compute the kernel function of a support vector machine. The implementation of the proposed model was carried out in Simulink, and simulation results showed that it had better performance in terms of speed and occupied area when compared to other solutions. To better evaluate its figure, a practical case of a polynomial kernel function was considered. Simulink and VHDL post-implementation timing simulations and measurements on FPGA confirmed the good results of the stand-alone accumulator.

## 1. Introduction

In recent years, the concept of a smart home has been extended from the simple automation and automatic control of the home appliances to a more complex management of the user interaction with several sensors and actuators deployed in the home environment in order to pursue the users’ wellbeing and energy sustainability [1,2,3,4,5,6,7,8,9]. The development of wearable sensors has expanded the possibilities available in this context, pushing research towards new solutions based on behavioral monitoring [10,11,12,13]. The role of wearable sensors in this framework is very wide, but recent research has focused on human activity recognition (HAR) as a new service to monitor the amount of activity for health purposes; this can be assessed and considered in order to early detect anomalies possibly relevant to users’ wellbeing [14,15,16,17].

The most advanced HAR algorithms are based on machine learning (ML) techniques, which are usually very computationally demanding [14]. The development of wearable devices leads to implementation of ML algorithms directly on board [18,19], allowing for the reduction of the amount of data to be transmitted, and with consistent advantages in terms of power consumption and system usability [14]. To address the issues related to the need for platforms with good computing capacity, instead of general-purpose processors, dedicated hardware architectures such as field programmable gate arrays (FPGAs) can be selected for the implementation of the algorithms [20,21,22,23]. This allows for the control of the resources needed for the task and to optimize the system for performance or physical size, depending on the use case. Recent advantages in FPGA technologies allow these platforms to also be used in applications with low cost [24] and/or low power consumption requirements [23].

The design of dedicated hardware architectures is traditionally done by using hardware description languages (HDL). However, as proven by different works [25,26,27], higher abstraction level frameworks can support the designer in helping to focus the attention on system functionalities and in reducing time-to-market. This is possible, for example, by using MATLAB/Simulink software [28]. A high-level approach can be developed, and the benefits of a model-based design can be exploited [29,30]. Moreover, with the dedicated HDL Coder tool, a HDL code can be automatically generated from the system block diagram and hence used to program the selected platform.

In this paper, we present the development of a model-based floating-point accumulator. To better study the performance of the designed model with respect to available solutions, it was applied to a practical case—a Simulink model-based kernel function conceived as the core of a support vector machine (SVM) classifier to be embedded in an FPGA-based wearable device. The SVM is a widely used algorithm for solving classification problems, also utilized in the field of HAR. The classification work consists in finding a line or a hyperplane that allows data to be divided into different regions. In the case of non-separable sample sets, the kernel function has to be introduced into the SVM algorithm [31]. Different approaches can be found in the literature—the linear, the polynomial, the Gauss radial basis, and others. A combination of them is often also proposed [32]. In the present work, the polynomial solution was adopted to evaluate the proposed accumulator because it has been recognized as the one with strong generalization capability [32]. The polynomial kernel function, as well as other kernels, involves the dot product of the input vectors, resulting in a sum of products to be implemented in arithmetic blocks. Among these, the accumulator has an important role. 

The simplest accumulator architecture can be designed by using an adder in which the first input receives the operand element and the second input is the feedback of the output [33]. It is worth noting that general FPGA-based SVM architectures deal with data with high dynamic range; thus, they are based on floating-point arithmetic, as this is the best solution with data with this requirement [34]. A typical approach when dealing with hardware floating-point arithmetic is to introduce pipelined architectures to reduce the critical path timing, potentially increasing the system clock frequency [35]. When used in the simple accumulator architecture described before, pipelined floating-point adders become critical. In fact, new input should be presented only when the output of the last addition can be fed back to ensure correct operation and avoid data hazards independently on the number and the length of the input vectors [33]. This would limit the applicability of the system, and different accumulator architectures should be individuated. However, when the boundary conditions allow this solution to be exploited, the latency of the whole accumulator for an input vector of *n* elements is T=np, where *p* is the length of the adder pipeline.

Considering model-based designs and, in particular, the Simulink environment, several accumulator blocks are already available. However, some of them are not suitable for the specific application due to incompatibility with the HDL Coder workflow (such as the *Cumulative Sum* block) or with the Floating-Point HDL library (as for the *Multiply-Accumulate* block). Some others (*Sum of Elements* and *Matrix Sum*) implement the HDL code as a binary tree or a linear chain of floating-point adders presenting all the input elements in parallel—since the complexity of these solutions grows with the number of inputs to accumulate, the amount of resources used when these blocks are exploited in this field should be evaluated [36]. Hence, in this paper, a possible alternative Simulink model for an accumulation circuit based on a floating-point pipelined adder and fully compatible with the HDL Coder workflow is presented. The paper is organized as follows: in Section 2 a review of the state-of-the-art accumulation circuits is presented. The architecture of the developed accumulator and kernel are introduced in Section 3, whereas in Section 4, tests are described and results are discussed. In Section 5, conclusions are drawn.

## 2. Related Works

To select the architecture to be implemented, a review of the state-of-the-art accumulation circuits was carried out. In reference [37], Luo and Martonosi present an architecture of an accumulator in which a floating-point pipelined adder is broken down into its mathematical operations (i.e., the ones involving the sign, mantissa, and exponent) and an internal feedback loop is introduced to embed the accumulation feature. This solution is reported as providing a minimal accumulation latency of $T=p+\left(n-1\right)+t_{norm}$, where $t_{norm}$ is the combinational logic delay of the last accumulation part of the architecture. However, the exact latency value cannot be determined a priori because it is strongly dependent on the target hardware architecture [37].

A similar approach is used by Nagar and Bakos in reference [38], in which the accumulation latency is independent from the hardware implementation, and $t_{norm}$ can be considered as a one clock cycle. However, as for reference [37], the model-based implementation of this solution implies the additional development of a new adder architecture in order to consider the required modification.

In reference [36], Zhuo et al. present two main architectures based on standard floating-point adders: the fully compacted binary tree (FCBT) and the single strided adder (SSA). FCBT is an accumulator derived from a binary adder tree in which the first level is replaced by one buffer and a single adder, and the rest of the levels are replaced by an additional adder shared by ⌈logn⌉−1 buffers. With the proper control logic, the system can perform the accumulation in T≤3n+(p−1)⌈logn⌉−3 for $n<n_{max}$, where $n_{max}$ is the maximum input vector length that the system has been designed to work with. Because two different floating-point adders were deployed, this solution turned out to be undesirable, as it requires large area resources [36]. 

To overcome this issue and to remove the $n_{max}$ limitation, the SSA architecture has been introduced. This architecture is based on a single adder, two buffers, and a control logic. With this system, the latency has proven to be $T\leq n+2p^{2}$. 

A different set of architectures are based on the work presented in [39]. Here, an implementation of an accumulator based on a standard pipelined floating-point adder is described. The input data vector is split in two different buffers and, at each clock cycle, one element from each buffer is given to the adder operands. Then, after *p* cycles, the vector of adder results are split in two halves again, which serve as the new input elements. This procedure is repeated until no other couples of operands are present in the buffers, meaning the accumulation has ended and the result is ready. This architecture was found to produce the accumulation result in T=(p−1)⌈logn⌉+3(n−1). The main limitation of this system is that only one input vector can be accumulated at a time, resulting in the fact that the subsequent vectors must wait for the current result to be produced before they can be processed.

The time and the resources needed to perform the accumulation are reduced in [40]. Compared to the work presented in [39], the input buffers are substituted by two multiplexers at the input of the adder. One multiplexer can switch between the input vector and a register holding the adder output, and the other can switch between a constant value and the direct adder output. With the proper control of the multiplexers and the register, the time needed to compute the accumulation is improved for n>p. Then, the resulted latency is

(1) $$T=\{\begin{array}{c} \left(p-1\right)\lceil \log n\rceil +3\left(n-1\right)\;\;\;\;\;\;\;\;\;\;,\;\;\;n\leq p\\ n+\left(p-1\right)\lceil \log p\rceil +4\left(p-1\right)\;\;,\;\;\;n>p \end{array}\;\;\;\;.$$

From this work, the total accumulation time of this circuits is found to be

(2) $$T=T_{f}+T_{m}+T_{d}\;\;,$$

where $T_{f}$ is the time needed for all the input elements to get inside the accumulator (feed phase), $T_{m}$ is the time needed to process all the partial results given by the couples of adder input operands (merging phase), and $T_{d}$ is the time needed for the last result to exit the adder pipeline (drain phase). It can be shown that this formula is applicable to every accumulator based on the architecture presented in [40]. Moreover, it can be easily observed that $T_{f}=n$ and $T_{d}=p-1$. 

In reference [41], an improved control algorithm (i.e., asymmetric method (AM)) for the merging time is presented. In this case, the merging time was found as

(3) $$T_{m}^{AM}=\{\begin{array}{c} n\lceil \log n\rceil -2^{\left\lceil \log n\right\rceil }+n+\left(k-n\right)\lceil \log n\rceil \;\;,\;\;\;n<p\\ p\lceil \log p\rceil -2^{\left\lceil \log p\right\rceil }+p\;\;\;\;\;\;\;\;\;\;\;\;\;\;\;\;\;\;\;\;\;\;\;\;\;\;\;\;\;\;\;\;\;\;\;,\;\;\;n\geq p \end{array}\;\;\;,$$

which shortened the total accumulation time by 3(p−1).

In reference [42], a modified AM is proposed, with an improvement for every n<2p:

(4) $$T_{m}^{AM}(n)=\{\begin{array}{c} T_{m}^{AM}\left(n\right)-p\;\;\;\;\;\;\;\;\;\;\;\;\;\;\;\;\;\;\;\;\;\;\;\;,\;\;\;n\leq p\;\;\\ T_{m}^{AM}\left(n\right)-p+1\;\;\;\;\;\;\;\;\;\;\;\;\;\;\;\;\;,\;\;\;n=p+1\;\;\;\;\\ T_{m}^{AM}\left(\left\lfloor n/2\right\rfloor \right)-D\left(\left\lfloor n/2\right\rfloor \right)\;,\;\;\;p+1<n<2p\\ T_{m}^{AM}\left(n\right)\;\;\;\;\;\;\;\;\;\;\;\;\;\;\;\;\;\;\;\;\;\;\;\;\;\;\;\;\;\;,\;\;\;n\geq 2p \end{array}$$

where *D* is a displacement function that compensates the irregular merging pattern that characterizes the control logic.

In reference [43], Tai et al. propose a modified version of [42], introducing the delayed buffering (DB) algorithm, in which the control logic can further reduce the merging time $T_{m}^{DB}$ in respect to $T_{m}^{MA}$ for certain input set lengths:

(5) $$T_{m}^{DB}=\{\begin{array}{c} p\lceil \log n\rceil +2^{\left\lceil \log n\right\rceil }+n-p\;\;,\;\;\;n\leq p\;\;\;\;\;\;\;\;\\ p\lceil \log p\rceil +2^{\left\lceil \log p\right\rceil }+n-p\;\;,\;\;\;n=p+1\\ pL-2^{L}+\lfloor G\rfloor -D+1\;\;\;\;\;\;\;,\;\;\;n>p+1 \end{array}\;\;\;\;,$$

where *L* and *G* are functions of *n* and *p*, which, as the *D* function, compensate the irregular merging pattern.

In reference [44], a solution requiring variable number of adders is presented—this increased the reuse and portability of the accumulator, but with higher occupied area. For example, for the area-efficient modular fully pipelined architecture (AeMFPA), two adders are required. More recently, reference [45] presents an accumulator circuit that can simultaneously add multiple independent vectors; however, the input buffer size is dependent on the number of the inputs, limiting the portability over different applications. Finally, in reference [46], a more flexible solution is reported—the core of the idea is a new state-based method (SBM) algorithm, a scheduling strategy for buffer management aiming at a lower latency and smaller area. 

In Table 1, a summary of the performance of the mentioned architectures is reported, along with some practical examples that were computed considering an adder latency of *p* = 11, as the latency of Simulink floating point adder intellectual property (IP), and an input length of *n* = 15, as well as an adder latency of *p* = 14 and *n* = 16 for comparison with the solution reported in [46].

As can be seen the system presented in [43], it offers the lowest latency for the accumulation of an input set of data. In this case, the total accumulation time depends on the input vector length with respect to the pipelined adder latency as expressed in Equation (6).

(6) $$T=T_{f}+T_{m}^{DB}+T_{d}=\{\begin{array}{c} n+p-1+p\lceil \log n\rceil +2^{\left\lceil \log n\right\rceil }+n-p\;\;,\;\;\;n\leq p\;\;\;\;\;\;\;\\ n+p-1+p\lceil \log p\rceil +2^{\left\lceil \log p\right\rceil }+n-p\;\;,\;\;\;n=p+1\\ n+p-1+pL-2^{L}+\lfloor G\rfloor -D+1\;\;\;\;\;\;\;,\;\;\;n>p+1 \end{array}$$

This model was exploited in the proposed model-based implementation and in the SVM kernel. It was fully tested in an FPGA implementation and, to validate the results, it was compared with the simple iterative accumulator solution [33], SBM [46], and the built-it *Sum of Elements* Simulink block. Then, the proposed accumulator was used in a model-based implementation of the SVM kernel function. 

## 3. Materials and Methods

### 3.1. Accumulator Architecture

**Figure 1:**
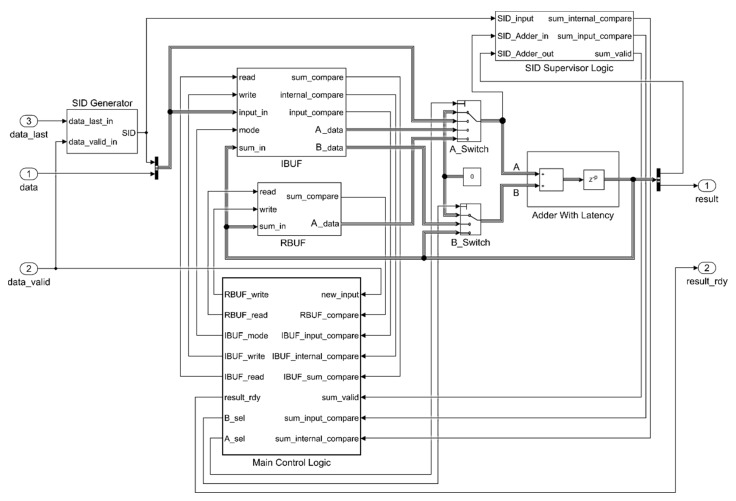
Simulink multi-set delayed buffering (DB) accumulator implementation.

In reference [43], two versions of the DB algorithm with different input processing properties are described. The first one, the single-set DB, is able to process one input vector at a time. If more than one vector has to be accumulated, each vector has to wait for the result of the previous one to be processed. The second algorithm is the multi-set DB, which is able to process a continuous stream of input vectors without the need to wait for the output results to be produced. An implementation of the single-set DB is presented in [28]. Because the data is processed in a streaming fashion in the SVM context, in this paper we focused on the multi-set DB version, although it required a more complex design with respect to the single-set design. In Figure 1, the proposed Simulink model-based accumulator is shown.

To design the proposed model, basic Simulink blocks were used. The core of the architecture is the adder that must handle floating point inputs. When dealing with floating point arithmetic, pipelined structures were introduced to ease the timing closure and achieve the desired operating frequency. In fact, a reduction in the total propagation delay, and then a higher clock frequency, can be obtained at the expense of an increase in the occupied area and in data path latency, due to the introduction of registers to segment the combinational logic. Pipelined adders can be modeled in Simulink as a cascade of an adder and a delay block; for this purpose, an *Adder With Latency* block was introduced. This configuration also allows for the configuration of the latency *p* of the adder with a customizable value. Moreover, in this implementation, the adder was set to manage input data with 32 bit length compatible with the IEEE 754 single format; however, if higher precision is requested, the adder can be set accordingly and the whole architecture automatically scales consequently. The remainder of the architecture features two multiplexers (modeled with Simulink Switch blocks *A_Switch* and *B_Switch*); two multiple-word registers (*Input Buffer* (*IBUF*) and *Result Buffer* (*RBUF*)); and a control logic that is composed of three blocks: *Set IDentification (SID) Generator, Adder Supervisor Logic*, and *Main Control Logic*. The *SID Generator* takes care of the tagging of the input elements to track each element of different sets. Each time the *data_last* flag is asserted together with the *data_valid* flag, the SID value is increased by one and it is merged into the internal bus together with the data value. Thus, all the data in the operands path are a pair of input data and a SID. The size of the counter (i.e., the maximum value of the SID) can be precisely set by knowing that, as found in [43], there cannot be more than ⌈5p/3⌉ sets at the same time inside the architecture. The *Adder Supervisor Logic,* instead, tracks all the SIDs to notify whether the current adder output is of the same set of any other set inside the adder pipeline (*sum_internal_compare*) or the current adder output is of the same set of the input (*sum_input_compare*) or, finally, a new adder output is produced (*sum_valid*). To achieve this result, the internal architecture exploits comparators and simple logic functions. The *Main Control Logic* is the core control unit of the system. The model-based implementation relies on the pseudocode presented in [43] and exploits full combinational logic. The inputs of the logic function are flags indicating relevant events (if new data are available, if a new sum is ready, etc.) and, at the output, produce the configuration setups for all the involved elements (i.e., IBUF, RBUF, A and B working modes, and when the output accumulation is ready). No sequential logic was used for this block, resulting in an output update rate independent from the system clock.

**Figure 2:**
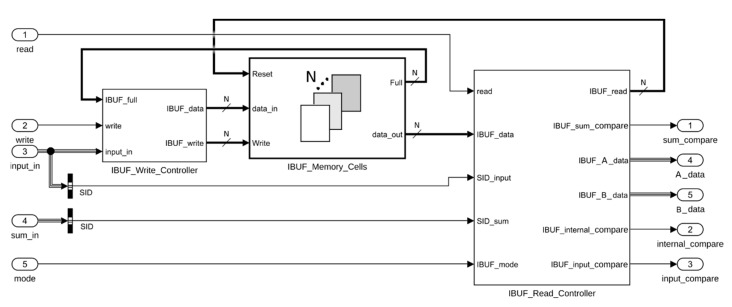
Architecture of the *Input Buffer* (*IBUF*) block.

As mentioned, in a multi-set DB version, two different buffers are needed. The *IBUF* buffer stores all the input elements that cannot enter the adder immediately because a couple of the same set (i.e., with the same SID) is not yet available. It is composed of an array of memory cells and two controllers, one for read and one for write operations. The model-based architecture is shown in Figure 2.

The memory cells array can store the data values along with their SIDs. The model-based implementation of this part exploits the *For Each* Simulink subsystem, which can scale and replicate its internal architecture (i.e., the single memory cell, in this case) based on a parameter N. According to the work presented in [43], N was set to ⌈p/2⌉ to guarantee no storage overflow. When a write operation is issued, the write controller drives the input data to the first empty cell of the array by setting the proper *IBUF_write* array value to 1.

The read controller takes as input the content of all the cells, the SIDs of the input and of the sum, the mode of operation, and the *read* flag. When a read operation is requested by setting the *read* bit, the content of one or two cells are presented at the *A_data* and *B_data* ports depending on the *mode* input signal. When *mode* is equal to 1, one value is read and is assigned to *A_data*, which is managed by the A-Switch (Figure 1) accordingly to the main control logic combinational function, and eventually set as input of the adder. In this case *B_data* value is kept un-set, leaving the other input of the internal accumulator adder decided by the main control logic through the B_Switch (Figure 1). If *mode* is equal to 2, a pair of data of the same set has to be read. The controller logic automatically selects the pair having the same and oldest SID, giving priority to the oldest accumulation result production. The read data are assigned to *A_data* and *B_data*. When *mode* is equal to 3, the behavior is specular to mode 1: the single read value is assigned to *B_data* and *A_data* is not used.

The remainder of the output signals serve as inputs for the *Main Control Logic* and are produced by combinational logic. In particular, the *sum_compare* and *input_compare* signals are produced by looking for the cells with the same *SID_sum* and the *SID_input*. The *internal_compare* signal is computed by comparing the internal content of the memory cells and it is used to notify if two or more memory cells hold data of the same set.

The purpose of the *RBUF* is quite similar, except that it holds all the adder outputs that cannot be re-introduced yet as inputs, as there are not a couple of operands with the same tag to be processed already. The implementation results in a subset of the architecture of *IBUF*. In fact, its input signals are only the read, write, and *data_in* values and its output signals are the *sum_compare* and *A_data* values. For this architecture, the value of N was set to ⌈2p/3⌉.

### 3.2. Kernel Architecture

In order evaluate the accumulation circuits described thus far, they were exploited in the development of a model-based design of a polynomial kernel. As defined in [47], the kernel equation is

(7) $$k\left(x,x^{\prime }\right)={\left(\langle x,x^{\prime }\rangle +c\right)}^{n}\;\;,$$

where $\langle x,x^{\prime }\rangle$ is the dot product between the input vector $x^{\prime }$ (also called the features vector) and the training input matrix (also called support vectors) x. As can be seen by Equation (7), the process involves a dot product, and hence an accumulation stage that directly affects the performance of the system.

To evaluate the optimal values for the parameters *c* and *n* in Equation (7), an offline training process was performed on the training set employed in [14], which contains the acquisitions of an inertial measurement unit (IMU) sampled at 50 Hz. From this training phase, the *n* and *c* parameters were found to be equal to 3 and 1, respectively, according to works dealing with similar problems [48,49]. Then, the resulting function can be expressed as

(8) $$k_{j}\left(x,x^{\prime }\right)={\left(\langle x,x^{\prime }\rangle +1\right)}^{3}={\left(\overset{N}{\underset{i=1}{\sum }}\left(x_{i}\cdot x_{ij}^{\prime }\right)+1\right)}^{3}\;\;,$$

where *N* is the length of the features vector *x*.

**Figure 3:**
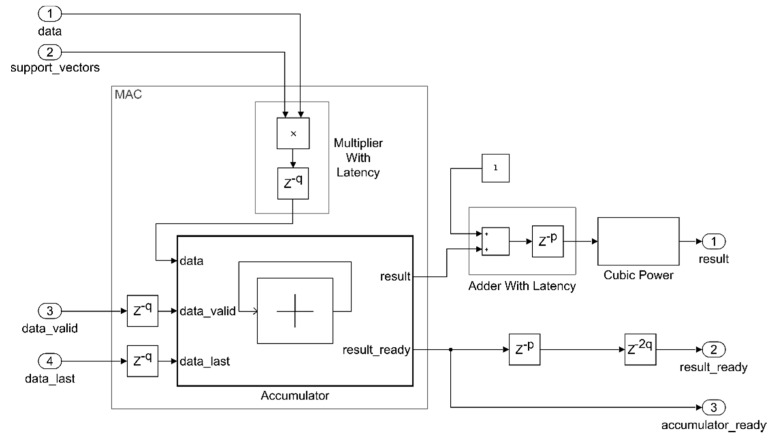
Simulink cubic kernel implementation.

In Figure 3, a model-based implementation of the cubic kernel is shown. It embeds a multiplier, an accumulator, and a cubic power block. It was tested with different architectures for the accumulator, as explained in the following sections. 

All the data flowing inside the kernel are in floating-point 32 bit format. As for the adder block described earlier, the multiplier block was also modeled with a Simulink floating point IP cascaded with a delay block in order to take into account the introduced latency (*q*). To synchronize all the data paths, several delay lines were introduced to compensate the latency of the mathematical operations. For example, as the cubic power block is implemented as a cascade of two multipliers, the required synchronizing delay on the *result_rdy* signal is two times the delay of a single multiplier (*2q*). The input scheduling should be tailored according to the selected accumulator architecture. 

## 4. Results and Discussion

### 4.1. Stand-Alone Model-Based Accumulator

To assess the performance of the proposed accumulator, Simulink simulations were carried out. The standard Simulink IP adder was exploited in the accumulator architecture. This block featured an internal latency of *p* = 11 cycles. Two mathematical series were exploited as input and the latency, and the correct accumulation results were evaluated. In particular, the inputs were the Euler’s number *e* and the Leibniz π mathematical approximation series, defined as

(9) $$e=\overset{\infty }{\underset{k=0}{\sum }}\frac{1}{k!}\;\;,$$

(10) $$\pi =\overset{\infty }{\underset{k=0}{\sum }}\frac{4\cdot {\left(-1\right)}^{k}}{2k+1}\;\;.$$

The series was generated in MATLAB environment as a 50 element vector for the Euler’s number series and as a 200 element vector for the Leibniz π series, in to obtain an approximation error lower than 0.5%. Then, the two vectors were imported in Simulink with the *From Workspace* block and presented as input to the accumulator. 

**Figure 4:**
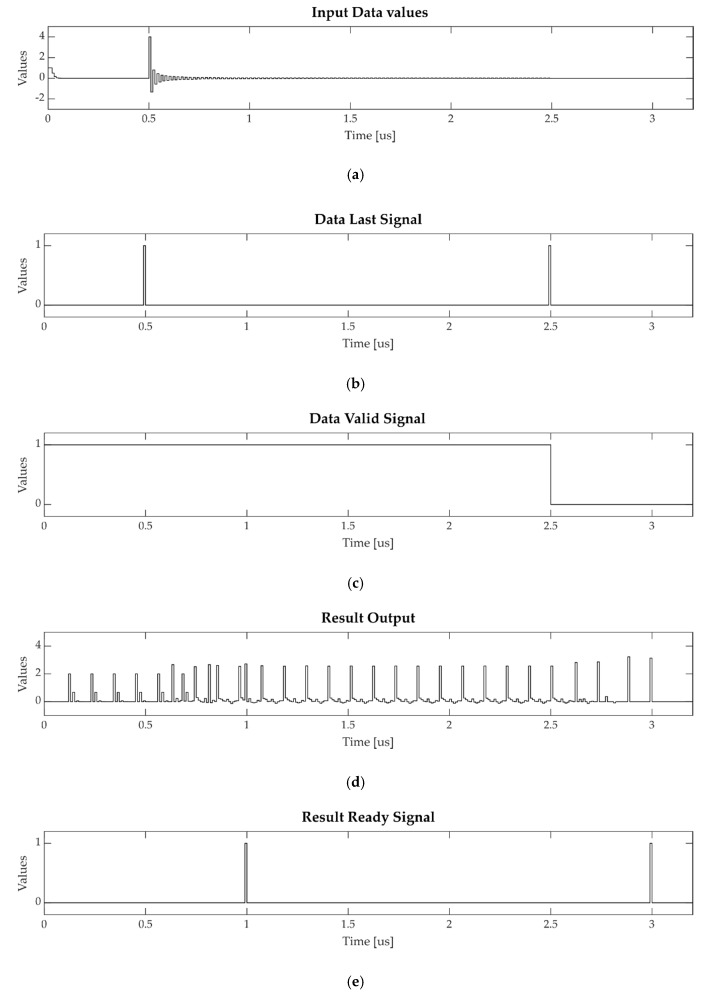
Input and output signals of the multi-set DB accumulator in Simulink simulation: (**a**) Input vector values, (**b**) *Data_last* signal, (**c**) *Data_valid* signal, (**d**) Accumulator output value, and (**e**) *Result_ready* signal.

In Figure 4, the results of the accumulation of two input vectors are shown.

The two vector series were presented to the input of the accumulator as a data stream, Euler’s series first (at t = 0μs), and then the Leibniz series (at t = 0.5 μs) (Figure 4a). The last element of a single vector was highlighted by the *data_last* signal (Figure 4b). The *data_valid* signal was high until a valid data is given to the accumulator input (Figure 4c). The accumulation outputs (Figure 4d) were evaluated when the *result_ready* signal was asserted (Figure 4e). From this simulation, the correctness of the results can be assessed. From Equation (6), considering a latency of *p* = 11 cycles and a simulation step of 10 ns, the first result was produced 99 cycles after the first input element. Then, the second result was produced 200 cycles after the first result. These time intervals can be verified from Figure 4.

Once the functionality of the accumulator architecture was verified, VHDL code was automatically generated from the Simulink model and used to synthetize the circuits in Xilinx Vivado software. Here, results were evaluated in terms of FPGA look-up tables (LUTs), flip-flops (FFs), and (Digital Signal Processor) DSP usage, and maximum achievable clock frequency. For this purpose and to show portability, two different platforms were considered: a Xilinx Artix-7 XC7A100T FPGA device along with Xilinx Vivado 2019.1 software and an Altera Cyclone 10 LP 10CL010 with Quartus 19.1. All the simulations and timing results were carried out considering a clock frequency of 100 MHz. In these experiments, a stream of 200 vectors, each one of 100 elements, was considered to highlight the capability of the models to process subsequent vectors in a short timeframe, without the need of complex input synchronization logic.

The performance of the proposed accumulator model was compared to that of the available Simulink solution. The Simulink IP block takes as input a set of data in parallel to perform the sum. If the input values are fed serially, an input buffer is needed to host all the elements. The time needed for this buffering stage is equal to the length of the input stream, and the length of the buffer represents the maximum vector length the system can accumulate. This, in a VHDL implementation, limits the input streaming vector length. During the VHDL generation process, the accumulator architecture is designed as a binary tree adder or a linear adder chain. For this comparison, the input buffer was set to 100 samples and the architecture to the one offering the lowest implementation resource usage, i.e., the linear adder chain. In Table 2, the post-implementation results for Xilinx are reported, whereas data for Altera are shown in Table 3. 

As can be seen from Table 2 and Table 3, the proposed accumulator outperformed Simulink IP in both area and time. In particular, from the area point of view, in Xilinx implementation, the proposed model used 2.6% of available LUTs and 0.97% of slice registers, whereas Simulink IP used 63.4% and 26.4%, respectively. Moreover, in the Altera implementation, although the quantity of logic elements (LEs) of the proposed accumulator corresponded to 24%, the Simulink IP could not be implemented; in fact, the occupied area saturated the resources, resulting in a 460% quantity of logic elements. For this reason, the achievable maximum frequency was not reported in this case. The advantage of the new model over the available IP appears evident. It is worth noting that, despite the low area occupied, the proposed solution does not require DSP slices, resulting in independence from the presence of these blocks in the selected platform, enhancing portability.

To evaluate the performance of the proposed model-based accumulator, once generated, in respect to other solutions, some comparisons were made with other accumulator architectures and available IPs: iterative accumulator, single-set DB, SBM, and Vivado floating-point accumulator IP. These architectures, Vivado IP in particular, are not suitable for automatic code generation; however, these data can give some information about the applicability of the whole process and can confirm the choice of the architecture. In Table 4, the Xilinx Artix 7 post-implementation results of the compared accumulator architectures are reported. 

The Xilinx Floating-Point IP is made of a fixed-point accumulator wrapped by floating-point conversions at the input and the output stages. To support the full precision and range of the 32 bits floating-point format, the internal fixed-point accumulator register must be correctly configured. The DSP slice usage was disabled to make a fair comparison with the proposed model. Moreover, the architecture optimization was set to produce the lowest latency—with this configuration, the internal fixed-point adder latency value resulted in 23 cycles. As can be seen from Table 4, the proposed model occupied less than a half in the area, without a significant difference in maximum frequency. Furthermore, the achievable frequency of the proposed accumulator is compatible with the maximum frequency allowed by the target FPGA.

Regarding the comparison with other architectures presented in the literature, the proposed accumulator outperforms the iterative and the single-set DB architectures in terms of latency needed to produce the result. It is important to note that this difference arises from the fact that the selected architecture is designed to process a stream of consecutive vectors, whereas both the iterative and the single-set DB solutions do not have this capability. The greater the number of vectors to be processed, the greater the latency associated with the latter two architectures. Furthermore, the buffers used for the management of the inputs synchronization must be carefully designed by considering the size of the vectors stream. SBM architecture performs well in terms of the occupied area. As a percentage, it occupies the 2.3% of the available LUTs and the 0.8% of the available slice registers. However, these numbers are close enough to that observed for the proposed model. The same goes for the maximum frequency, with a slight advantage for the selected accumulator. The new model also presents good results in latency, confirming the correct choice of the architecture also compared to the newer solutions presented in the literature.

### 4.2. Evaluation of the Proposed Model in a Practical Context: The Case of SVM Kernel Function

To frame the accumulator performance in a practical context, we evaluated it in the design of a cubic kernel function architecture conceived for an SVM applied to HAR. The inputs for the kernel were computed from datasets described in [14], where nine different daily activities have to be recognized. Data from a 9 degree of freedom (DoF) inertial measurement unit (IMU) were collected and processed, resulting in a dataset of 15,616 instances. This dataset was divided into a training set, used to train the SVM algorithm, and a test set, used in the inference phase. The support vectors $x^{\prime }$, described in Equation (8), were computed during the training phase. In particular, each instance was labelled as belonging to an activity and was processed to extract a vector of nine features representing statistical values (mean, standard deviation, etc.) of the nine DoF data, resulting in a vector of 81 elements. The support vector in Equation (8) refers to a binary problem—as this dataset refers to a multidimensional problem, 36 support vectors are need to resolve the whole classification. In this experiment, a single support vector of 207 × 81 elements related to a single binary problem was selected and used as *support_vectors* input of the presented kernel architecture. The same statistical elaboration was applied to data in the test set—one vector of 81 elements, representing one instance of the test set, was exploited as the *data* input of the kernel architecture.

The kernel function was designed as a model-based block in Simulink. For the accumulation process, we compared our solution with the Simulink IP. An HDL code was generated and implemented on the same Xilinx Artix-7 FPGA exploited for the stand-alone accumulators, with a clock frequency of 100 MHz.

In this practical evaluation, other than Simulink and VHDL post-implementation simulations, measurements on hardware implementation were performed. 

Simulink simulations were performed to compare the proposed model latency with the ones of the kernel implementation with Simulink IP. In both the implementations, standard Simulink floating-point adder and multiplier IP were exploited. Similar to the adder IP, which had an already mentioned latency of *p* = 11, the internal latency of the multiplier IP was found as *q* = 6.

**Figure 5:**
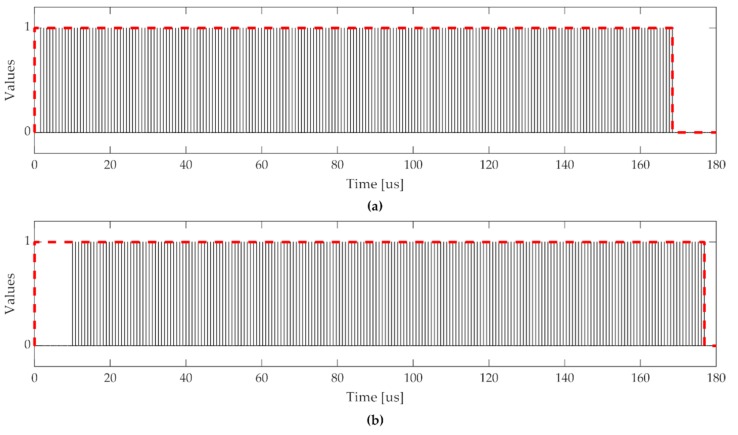
Kernel performance in Simulink simulations: (**a**) Kernel with proposed accumulator, (**b**) Kernel with Simulink IP accumulator.

In Figure 5, Simulink simulations are shown, in which the *result ready* signals are plotted. The dashed line refers to the time taken to complete the processing of the dot product of the whole 207 × 81 support vectors and the 1 × 81 data vector. In the case of the proposed model (Figure 5a), the time needed to accumulate the first vector at the input was equal to 161 cycles. Then, 206 × 81 cycles were needed for the remaining vectors. Considering a clock frequency of 100 MHz, this corresponded to 168.5 μs.

In the case of Simulink IP (Figure 5b), with an input stream of 81 elements and p=11, a total time of 891 cycles were required to obtain the correct accumulation, along with 111 cycles for the remainder of the kernel operations, starting from when the first element was available. Hence, the kernel processing for the first vector took 1002 cycles, and then 206 × 81 cycles were needed to complete the processing, corresponding to 176.9 μs.

**Figure 6:**
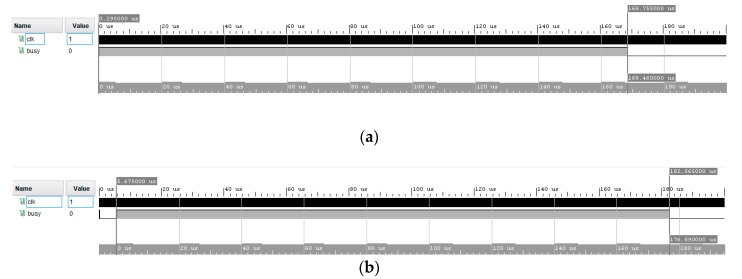
Xilinx Vivado post-implementation results of the kernel with (**a**) Kernel with proposed accumulator, (**b**) Kernel with Simulink IP accumulator.

The kernel models’ VHDL codes were automatically generated, and performance was evaluated in Vivado environment in terms of resources usage and maximum achievable frequency. Moreover, the latencies resulting from Simulink were verified in the Vivado post-implementation timing simulations. A busy signal was configured in order to be high from the first element presented at the input to the last kernel output produced. Examples are shown in Figure 6.

Performance in terms of resources usage, maximum achievable frequency, and latency are summarized in Table 5.

The resulted latencies confirmed the Simulink simulations and the results on the stand-alone accumulators. The proposed model definitely performed better in terms of occupied area—it used only 5.2% of the available LUTs and 2.2% of the available registers for the whole kernel. Contrarily, the Simulink IP appeared critical in this context, with 65% and 27% of the LUTs and registers, respectively. Considering that many other logic blocks need to be instantiated together with the kernel in a complete SVM implementation, our solution appears a possible valid approach in this context. Moreover, it is worth noting that in wearable sensors, low power consumption has particular relevance. With the technology advancement in the FPGA field, as already mentioned, many low power models have been made available and can be exploited in this context, even considering floating point arithmetic [23]. The lowest power platforms have generally a low number of resources available; for this reason, the occupied area aspect is of utmost importance in these kinds of applications.

Although the maximum operating frequency was the same for both solutions, the resulting latency for our model was definitely lower.

**Figure 7:**
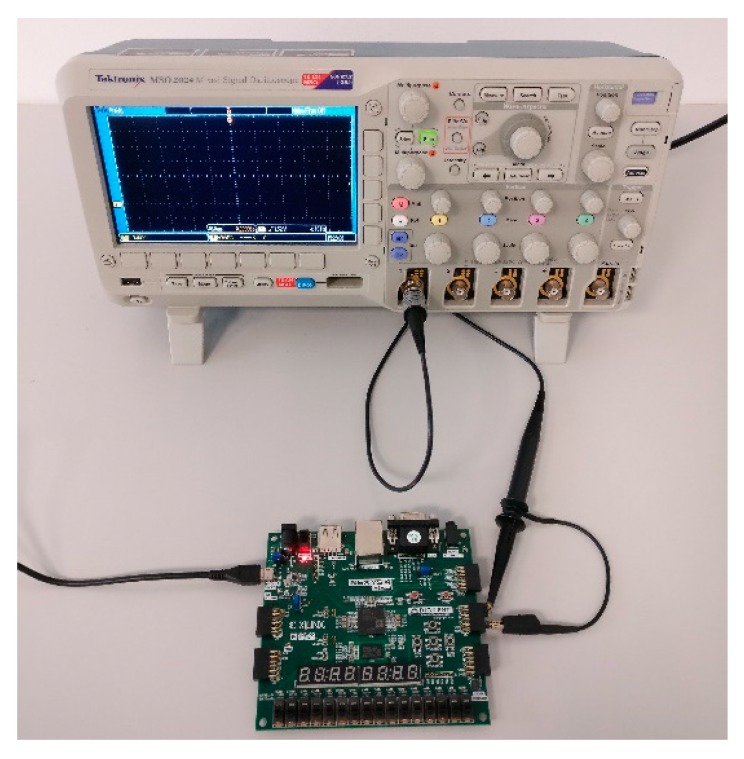
Experimental setup for the hardware measurement.

To further confirm the simulation values, the FPGA was configured with the generated code and the performance was measured directly on hardware. The busy signals were measured using a Tektronix MSO 2024 oscilloscope. In Figure 7 the experimental setup is shown.

**Figure 8:**
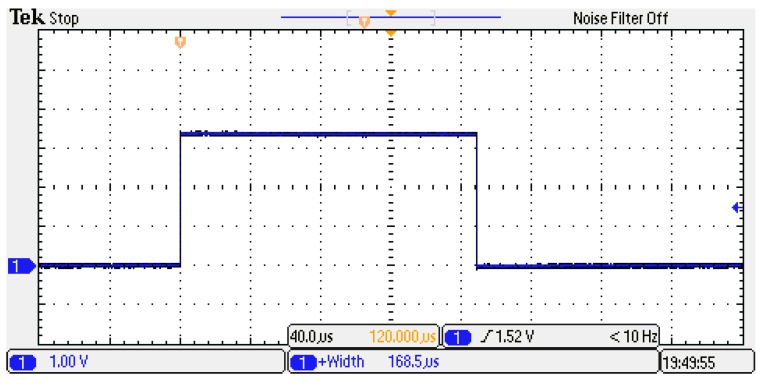
Measurement of the processing time of the kernel with the proposed accumulator implemented on the FPGA.

**Figure 9:**
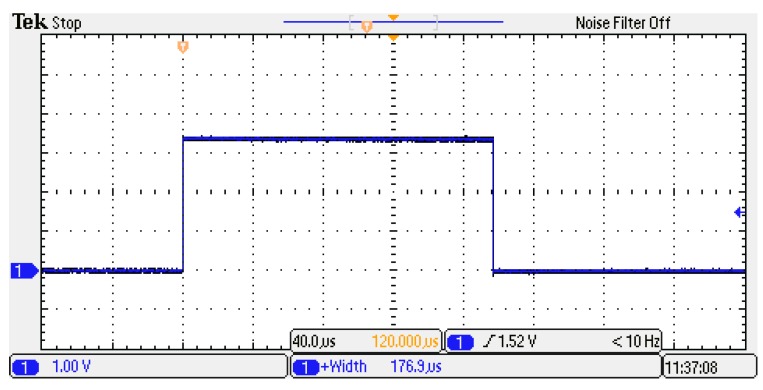
Measurement of the processing time of the kernel with Simulink IP implemented on the FPGA.

Results are reported in Figure 8 and Figure 9, which are related to the proposed architecture and the Simulink IP, respectively.

Measurements confirm the latencies of the simulations and the correctness of the result—the difference between the two processing times was of about 8.4 μs, which corresponds to 840 clock cycles. As can be seen from Table 5, this result corresponds with the difference in latency of the two solutions.

## 5. Conclusions

In this paper, a floating-point Simulink model-based accumulator architecture was presented. The functionality of the proposed accumulator was first tested with behavioral simulation in the Simulink environment. The tests were carried out using two mathematical series vectors as inputs. Results show the correct output accumulation values for both the series. Then, VDHL code was automatically generated and performance was assessed with post-implementation timing simulations on two different target FPGAs, a Xilinx Artix 7 and an Altera Cyclone 10 LP, in order to demonstrate portability. Results were compared with available Simulink IP supporting HDL code generation, demonstrating a significant reduction of about 95% in both area and time. Other solutions presented in the literature [28,33,46] and Vivado IP were compared, as well as demonstrating the applicability of the HDL code generation process and to confirm the choice of architecture. To frame the accumulator performance in a practical context, we evaluated it in the design of a polynomial cubic kernel function architecture conceived for an SVM applied to HAR. Additionally in this context, better performance was confirmed, greatly reducing the occupied area and making the solution particularly attractive for implementation in the context of wearable sensors, in which low resource platforms are usually exploited. The simulation results were also validated with hardware measurements on the target FPGA.

## Figures and Tables

**Table 1:** State-of-the-art hardware accumulator architectures.

Method	Accumulator Latency		
	Generic	p=11, n=15	*p* = 14, *n* = 16
SSA [36]	$\leq n+2p^{2}$	257	408
FCBT [36]	≤3n+(p−1)⌈logn⌉	85	100
AM [41]	$n+p-1+T_{m}^{AM}$	64	83
MA [42]	$n+p-1+T_{m}^{MA}$	58	71
A2eMFPA [44]	n+p⌈logp+2⌉	81	100
[45]	$n+T_{m}^{AM}+\lceil p/2\rceil$	60	104
SBM [46]	not available	-	75
DB [43]	$n+p-1+T_{m}^{DB}$	57	71

**Table 2:** Simulink accumulator resource usage, maximum frequency, and latency on Xilinx Artix 7.

	Proposed Model	Simulink IP
Slice LUTs	1643	40198
Slice registers	1239	33450
DSPs	0	0
BRAM	0	0
Fmax (MHz)	105	109
Latency (cycles)	49	989

**Table 3:** Simulink accumulator resource usage, maximum frequency, and latency on Altera Cyclone 10 LP.

	Proposed Model	Simulink IP
Logic Elements	2483	47430
DSPs	0	0
Memory (bits)	154	436648
Fmax (MHz)	108	N.A.
Latency (cycles)	49	989

**Table 4:** Post implementation accumulator resource usage, maximum frequency, and latency on Xilinx Artix 7 FPGA.

	Proposed Accumulator	Single-Set DB [28]	Iterative [33]	SBM [46]	Vivado IP
Slice LUTs	1643	749	658	1411	3245
Slice registers	1239	811	534	1027	3120
DSPs	0	0	0	0	0
BRAM	0	8.5	8.5	0	0
Fmax (MHz)	105	112	126	102	134
Latency (cycles)	49	9800	200,200	54	23

**Table 5:** Post implementation kernel resource usage and maximum frequency on Xilinx Artix 7 FPGA.

	Proposed Accumulator	Simulink IP
Slice LUTs	3266	41,354
Slice registers	2791	34,700
DSPs	3	3
BRAM	0	0
Fmax (MHz)	106	106
Latency (clock cycles)	161	1002
